# Bioactive, Degradable and Tough Hybrids Through Calcium and Phosphate Incorporation

**DOI:** 10.3389/fmats.2022.901196

**Published:** 2022-07-01

**Authors:** Francesca Tallia, Hung-Kai Ting, Samuel J. Page, Joshua P. Clark, Siwei Li, Tian Sang, Laura Russo, Molly M. Stevens, John V. Hanna, Julian R. Jones

**Affiliations:** 1 Department of Materials, Imperial College London, London, United Kingdom; 2 Department of Physics, University of Warwick, Coventry, United Kingdom; 3 Department of Bioengineering, Imperial College London, London, United Kingdom; 4 Institute of Biomedical Engineering, Imperial College London, London, United Kingdom; 5 Department of Biotechnology and Biosciences, University of Milano-Bicocca, Milan, Italy; 6 CÚRAM, SFI Research Centre for Medical Devices, National University of Ireland Galway, Galway, Ireland

**Keywords:** sol-gel hybrid, bone regeneration, bioactive, cyclic loading, calcium methoxyethoxide

## Abstract

We report the first inorganic/organic hybrids that show outstanding mechanical properties (withstanding cyclic loading) and bone bioactivity. This new hybrid material may fulfil the unmet clinical need for bioactive synthetic bone grafts that can withstand cyclic loading. A SiO_2_/PTHF/PCL-diCOOH sol-gel hybrid system, that combined inorganic and organic conetworks at the molecular level, previously demonstrated unprecedented synergy of properties, with excellent flexibility and promoted formation of articular cartilage matrix *in vitro*. Here, for the first time, calcium and phosphate ions were incorporated into the inorganic component of the hybrid network, to impart osteogenic properties. Calcium methoxyethoxide and triethyl phosphate were the calcium and phosphate precursors because they allow for incorporation into the silicate network at low temperature. The hybrid network was characterised with ATR-FTIR, XRD and solid-state Nuclear Magnetic Resonance, which proved calcium and phosphate incorporation and suggested the Ca^2+^ ions also interacted with PCL-diCOOH through ionic bonds. This resulted in an increased strength (17–64 MPa) and modulus of toughness (2.5–14 MPa) compared to the original SiO_2_/PTHF/PCL-diCOOH hybrid material (which showed strength of ~3 MPa and modulus of toughness of ~0.35 MPa), while also maintaining the ability to withstand cyclic loading. The presence of calcium and phosphates in the silicate network resulted in a more congruent dissolution of the inorganic and organic co-networks in TRIS buffer. This was shown by the presence of silicon, calcium and phosphate ions along with PCL in the TRIS buffer after 1 week, whereas Ca-free hybrids mainly released PCL with negligible Si dissolution. The presence of calcium and phosphates also enabled deposition of hydroxycarbonate apatite following immersion in simulated body fluid, which was not seen on Ca-free hybrid. All hybrids passed cell cytotoxicity tests and supported preosteoblast cell attachment. The phosphate-free hybrid showed the best mechanical behaviour and supported better cell attachment, spreading and potentially differentiation of cells. Therefore, the SiO_2_-CaO/PTHF/PCL-diCOOH hybrid represents a promising biomaterial for use in bone regeneration.

## Introduction

A sol-gel hybrid material has co-networks of inorganic and organic components that interact at the molecular level and are indistinguishable above the nanoscale (Novak, 1993; Jones, 2013). Hybrid materials have many potential applications (Sanchez et al., 2005; Sanchez et al., 2011; Kickelbick, 2014). The aim of producing hybrid biomaterials for bone regeneration is to attain synergistic properties of their organic and inorganic components, while maintaining bioactivity (bone bonding and osteogenic properties) of the inorganic component. The nanoscale interactions within hybrids make them new single-phase materials that overcome the issues typically observed in conventional composites, such as masking of the dispersed phase, incongruent degradation and tailorable mechanical properties (Jones, 2013; Tallia et al., 2018).

Currently, a device does not exist that can share load with the host bone, stimulate bone regeneration and biodegrade at a controlled rate. Bioactive glasses stimulate bone growth (Hench et al., 1971; Hench, 2006) but their inherent brittleness prevents their use in load-bearing sites, such as large or segmental bone defects (Valliant and Jones, 2011; Jones, 2013). Sol-gel hybrids have potential of increased toughness by introducing a polymer into a silica sol (Mahony et al., 2010; Valliant and Jones, 2011; Poologasundarampillai et al., 2012; Jones, 2013; Chung et al., 2016; Maçon et al., 2016; Tallia et al., 2018). The polymer molecules can entangle with the silicate network as it forms (Valliant and Jones, 2011; Jones, 2013). A key factor for obtaining hybrids with congruent and tailorable properties is ensuring strong covalent bonding between the organic and the inorganic networks, alongside weaker interactions such as hydrogen bonds (Poologasundarampillai et al., 2010). The covalent bonding is usually achieved by adding a so-called “coupling agent”, an organosilane molecule that possesses trialkoxysilyl moieties to co-condense with the silica network on one side and an organic tail used to covalently link the organic chains on the other side (Mahony et al., 2010; Poologasundarampillai et al., 2010; Valliant and Jones, 2011; Jones, 2013). Such hybrids are classified as Class II, when the organic component is pre-polymerised, and Class IV when the organic component is polymerised within the sol-gel reaction *in situ* (Novak, 1993; Jones, 2013; Tallia et al., 2018). The hybrid sol can be processed to obtain 3D porous scaffolds for tissue regeneration. The scaffold should have the appropriate balance between interconnected porosity, pore size, inherent mechanical properties, and bioactivity to support cell migration and promote tissue regeneration at the same rate of scaffold biodegradation and to provide mechanical stability and support while the scaffold degrades and the new tissue grows (Jones, 2009; Martin et al., 2012).

Previously, a hybrid material of SiO_2_ with polytetrahydrofuran (PTHF) and polycaprolactone as organic components and (3-glycidoxypropyl) trimethoxysilane (GPTMS) as the coupling agent was proposed by Tallia *et al*., with properties suitable for articular cartilage regeneration (Tallia et al., 2018). This resulted from innovative synthesis combining silica sol-gel synthesis with *in situ* cationic-ring opening polymerisation. GPTMS was added to oxidised PCL (PCL-diCOOH) that was dissolved in tetrahydrofuran (THF); when boron trifluoride diethyl etherate (BF_3_·OEt_2_) was added as a catalyst to open the GPTMS epoxide ring, unexpected ring-opening polymerisation of THF led to the formation of PTHF as second organic component; hydrolysed TEOS was then added to form the silica network that co-condensed with the GPTMS inorganic moieties, ensuring a covalent bond between the three components of the hybrid system. The resulting SiO_2_/PTHF/PCL-diCOOHhybrid material showed unprecedented mechanical properties, which are high flexibility and ability to recover deformation (“bouncing”), but also autonomous and intrinsic self-healing ability. These properties were never observed in Bioglass-derived biohybrids before. The hybrid sol was tailored such that it could be directly 3D printed into porous scaffolds (Tallia et al., 2018). Culturing human bone marrow stem cells in scaffolds with 200 μm channels, in chondrogenic media, provoked differentiation into a chondrogenic lineage, producing articular cartilage-like matrix, whereas 500 μm channels enabled the stem cells to flatten and produce collagen I matrix (Li et al., 2020).

For bone regeneration, stiffer but tough materials are needed and the bioactivity of bioactive glass is required for bone bonding and osteogenic stimulation (Jones, 2009). In the SiO_2_/PTHF/PCL-diCOOH hybrid, the inorganic component was previously only silica. Here, the aim was to replicate the composition of a bioactive glass, obtaining apatite layer formation through dissolution of Ca and P species from the silicate. The osteogenic properties of bioactive glasses have been attributed to the dissolution products stimulating osteoblasts (Xynos et al., 2001; Hench and Polak, 2002; Naruphontjirakul et al., 2018) at the genetic level and provoking differentiation of stem cells (Bosetti and Cannas, 2005; Naruphontjirakul et al., 2019) down an osteogenic route. The key dissolution products are soluble silica and calcium ions (Tsigkou et al., 2009), but introducing calcium into the inorganic (silicate) network of hybrid is a challenge as the soluble calcium salts used in solgel glass synthesis (e.g., calcium nitrate tetrahydrate) require heating above 400°C for calcium to enter the silicate network and become a network modifier (Skipper et al., 2005; Lin et al., 2009). Hybrids must be dried at low temperatures to avoid damaging the polymer and the requirement for heating to enable calcium incorporation and removal of toxic by-products has been neglected in many studies (Jones, 2013).

Early hybrid formulations used calcium salts and *in vitro* apatite formation was observed after 1 day in simulated body fluid (SBF) studies, but the calcium was not incorporated into the silicate network. Examples are the Class II glass/PCL hybrids by Rhee *et al*., which were synthesised using (3-isocyanatopropyl) triethoxysilane (ICPTS) as a coupling agent and calcium nitrate as the calcium source (Rhee et al., 2004; Yoo and Rhee, 2004; Yoo et al., 2007). Similar apatite formation after 1 day immersion in SBF was observed on Class II hybrids with PTHF as the organic component, ICPTS as coupling agent and calcium nitrate (Miyata et al., 2002; Miyata et al., 2004) or calcium chloride (Koh et al., 2010) as calcium source. However, the hybrids were only heated to 60°C so calcium was not incorporated in the silica network and toxic nitrate residues may not have been removed.

Calcium alkoxides, such as calcium methoxyethoxide (CME) and calcium ethoxide (CE), have been proven to be excellent calcium source alternatives since they allow for the Ca incorporation into the silica network at room temperature (i.e., ≤60°C) without affecting the organic phase (Manzano et al., 2006; Yu et al., 2012; Dieudonné et al., 2014). The hypothesis is that the CME undergoes hydrolysis when added to the hydrolysed TEOS sol, allowing the incorporation of calcium into the wet gel. The silica network then continues to cross-link during ageing and drying, maintaining the calcium in the network (Yu et al., 2012). CME was successfully used as calcium source in SiO_2_-CaO/poly(γ-glutamic acid) hybrids (Poologasundarampillai et al., 2014) and SiO_2_-CaO/polyethylene glycol bulk hybrids (Li et al., 2015), which both showed apatite formation after immersion in SBF for 3 days (Poologasundarampillai et al., 2014) and 24 h (Li et al., 2015) respectively, and promising cell response: SiO_2_-CaO/poly(γ-glutamic acid) hybrids were not toxic to the human bone marrow derived mesenchymal stem cells (hMSCs), which grew on the hybrids over the period of 7 days (Poologasundarampillai et al., 2014); good viability and proliferation of MC3T3-E1 cells were observed in presence of SiO_2_-CaO/polyethylene glycol hybrids (Li et al., 2015). The polyethylene glycol hybrid showed higher deformability than the poly(γ-glutamic acid) hybrid, but behaviour under cyclic loading was not reported. Calcium and silica containing PLLA sol-gel hybrid fibremats were successfully produced via modified dual syringe reactive electrospinning, using CME as the calcium source, however, it was a Class I hybrid and the fibre structure cannot be used in load-bearing skeletal regions (Poologasundarampillai et al., 2011).

Calcium ethoxide was also explored as alternative calcium source alkoxide in Class II silica-gelatin hybrids with GPTMS as coupling agent (Dieudonné et al., 2014; Lao et al., 2016). However, calcium alkoxides are highly sensitive to water and the choice of water-soluble gelatin as polymer component limits the synthesis process as a co-solvent must be used in combination with water and careful control of the process is required to avoid too fast gelation and consequent uneven distribution of calcium within the network.

None of the aforementioned hybrids contained phosphates in the glass component, which could have an improved effect on the bioactivity and on the biodegradation. Phosphate incorporation may also affect calcium incorporation as calcium has a higher affinity to coordinate phosphate rather than silicate, so the presence of phosphates could make less calcium ions available to modify the silicate network (Tilocca and Cormack, 2007). Triethyl phosphate (TEP) is a conventional P precursor used in sol-gel glass chemistry (Tallia et al., 2014; Ren et al., 2017; Ting et al., 2017), as it can be hydrolysed in aqueous solution to form orthophosphate ions (PO_4_^3-^), which are then charge balanced with calcium ions. When there is excess water in the sol, the orthophosphate units can form hydroxyapatite crystals in the glass (Ting et al., 2017). When using TEP for sol-gel glass synthesis, if the phosphate is below 8 mol%, it remains as orthophosphate units, but if the phosphate content is increased above 8 mol%, phosphate glass networks form (Ting et al., 2017). We hypothesise that CME and TEP can be added to the sol-gel hybrid synthesis to introduce Ca and P_2_O_5_ into the inorganic component of the hybrid.

Here, we present novel SiO_2_-CaO-P_2_O_5_/PTHF/PCL-diCOOH hybrids with potential for applications that experience cyclic loading, and investigate the effects of the addition of calcium (from CME) and phosphates (from TEP) into the SiO_2_/PTHF/PCL-diCOOH hybrid system (Tallia et al., 2018) to transform it into a hybrid material suitable for bone regeneration.

## Materials and Methods

### Materials

All chemicals were purchased from Sigma-Aldrich (UK) and VWR (UK) and all cell culture reagents were obtained from Invitrogen (UK) and Sigma-Aldrich unless specified otherwise. Reactions were carried out using commercially available starting materials and solvents without any further purification.

### Preparation of Calcium Methoxyethoxide (CME)

CME was prepared following the method described by Pickup et al. (2009) wherein 4 g of calcium pieces (<1 cm, 99%) were reacted with 96 ml of anhydrous 2-methoxyethanol under argon atmosphere at 80°C for at least 24 h. Then the resultant solution was centrifuged at 6,000 rpm for 15 min to remove unreacted calcium metal. The concentration of CME solution used in this work was 1 M. This was determined by transferring 1 ml of the CME solution into a platinum crucible and heating it to 1,050°C for 12 h, in order to evaporate all the solvent and convert CME to CaO. The concentration of CME in the resultant solution was then calculated as ratio between the mass of CaO and the molecular weight of CaO.

### PCL Oxidation

Polycaprolactone diol (PCL-diol, M_n_ 530 Da) was oxidised with 2,2,6,6-tetramethyl-1-piperidinyloxy (TEMPO) into the corresponding dicarboxylic acid (PCL-diCOOH) as described by Tallia *et al*. (Tallia et al., 2018).

### Preparation of SiO_2_-CaO-P_2_O_5_/PTHF/PCL-diCOOH Hybrid Monoliths

The inorganic glass sol and organic sol were prepared separately based on the process described previously (Tallia et al., 2018) for preparing Ca-free and P-free SiO_2_/PTHF/PCL-diCOOH hybrids, but CaO and P_2_O_5_ were incorporated into the SiO_2_ network to produce bioactive glass compositions using CME and TEP, respectively. The inorganic glass sol was first prepared by mixing 2.14 ml (2 g, 9.6 mmol) of TEOS, with the corresponding amount of CME and TEP, under continuous stirring at room temperature in a sealed polytetrafluoroethylene (PTFE) container for at least 3 h. Four molar ratios were prepared (Table 1): 1) TEOS/CME = SiO_2_/CaO = 60/40 mol%; 2) TEOS/CME/TEP = SiO_2_/CaO/P_2_O_5_ = 60/36/4 mol%; 3) TEOS/CME/TEP = SiO_2_/CaO/P_2_O_5_ = 60/32/8 mol%; 4) TEOS/CME/TEP = SiO_2_/CaO/P_2_O_5_ = 60/28/12 mol%.

Regarding the preparation of the organic precursor solution, 500 mg (0.94 mmol, TEOS/PCL-diCOOH = 80/20 wt%) of PCL-diCOOH were dissolved in 5 ml of tetrahydrofuran (THF, concentration of 100 mg ml^−1^), then 446 mg of GPTMS (417 μL, GPTMS:PCL-diCOOH = 2:1 M ratio) were added into the solution. After 10–15 min of stirring, 67 mg of BF_3_·OEt_2_ (58.2 μL, BF_3_·OEt_2_:PCL-diCOOH = 1:2 M ratio) were added into the resultant solution as a catalyst and left to mix for 90 min.

All hybrids were made with a TEOS/PCL = 80/20 wt%. Here, the hybrid samples were named after the inorganic glass compositions incorporated into the hybrids, as summarised in Table 1. SiO_2_/PTHF/PCL-diCOOH hybrids were used as reference materials in the characterisation studies, termed 100S-CL (previously termed Si80-CL by Tallia *et al*. (Tallia et al., 2018)). New hybrid compositions 60S(40-x)CxP-CL were synthesised by introducing the organic sol drop-wise into inorganic sols of 60 mol% SiO_2_, x mol% of P_2_O_5_ and (40-x) mol% of CaO. The resulting hybrid sol was left stirring for 1 h (until a homogenous solution was formed) before adding deionised water (TEOS:H_2_O = 1:2 M ratio) and 2 M nitric acid (1/6 volume of water).

Due to the high reactivity of CME towards water, the resultant hybrid sol gelled rapidly after water and 2M nitric acid addition, therefore the hybrid sol was transferred into cylindrical PTFE moulds (Ø = 15 mm; h = 25 mm) just before it gelled (usually within 30 min after the addition of water and nitric acid). The PTFE moulds were then sealed in a polymethylpentene (PMP) mould for ageing at 40°C for 3 days. Since the hybrids might crack if they are dried too fast, 1.5–2 ml of 70% aqueous ethanol was added in the PMP mould to enable a slow and controlled drying step. The samples were dried with gradual loosening of the lid, first at 40°C for ~2 weeks (until all the aqueous ethanol evaporated), then transferred to the 60°C oven until completely dry (~1 week). The dimensions of the final dried samples were determined by the shrinkage inherent in the sol-gel synthesis.

### Characterisation of Hybrid Networks

The final inorganic/organic (I/O) ratio was determined through thermogravimetric analysis (TGA) for each hybrid composition. The weight loss was due to the burning-out of the total organic content given by the sum of PCL-diCOOH, the organic portion of GPTMS introduced in the starting solution and the PTHF generated by *in situ* CROP; the inorganic content consisted of glass and the silica derived from GPTMS. DSC/TGA (Netzsch Jupiter STA 449C) was used on powders (10–15 mg), ground with a pestle and mortar and weighed out into a platinum crucible. The range of analysis was 20–750°C with a heating rate of 10°C min^−1^ in an atmosphere of continuously flowing air.

Further characterisation of the hybrid network involved ATR-FTIR (Fourier-Transform Infrared Spectroscopy in Attenuated Total Reflectance mode), XRD (X-Ray Diffraction) and solid-state NMR (Nuclear Magnetic Resonance) on samples manually ground to powder form with a pestle and mortar. ATR-FTIR was run using a Thermo Scientific Nicolet iS10 FTIR equipped with Smart Golden Gate for Single-Reflection Diamond ATR Analysis with OMNIC software; for each sample 64 scans at a resolution of 4 LP mm^−1^ were taken to form spectra in absorbance between the range 4,000–400 cm^−1^. XRD was carried out with a desktop diffractometer, Bruker D2 PHASER, using a step scanning with Cu radiation, at 30 kV and 10 mA, with 0.030° 2θ step size and a count rate of 0.5 s per step, from 2θ of 6° to 70°.

All solid state ^29^Si Magic-Angle-Spinning Nuclear Magnetic Resonance (MAS NMR) measurements were performed at 7.0 T using a Varian/Chemagnetics InfinityPlus spectrometer operating at Larmor frequencies of 59.6 MHz using a Bruker 7 mm HX probe which enabled a MAS frequency of 5 kHz to be implemented throughout. For the ^29^Si single pulse MAS NMR experiments, pulse time calibration was performed on kaolinite from which a π/2 pulse time of 5.5 μs was measured. All measurements were undertaken with a π/3 tip angle, a delay of 240 s, and a heteronuclear ^1^H/^29^Si decoupling field strength of 60 kHz during data acquisition. All ^29^Si chemical shifts were reported against the IUPAC recommended primary reference of Me_4_Si (1% in CDCl_3_, δ = 0.0 ppm), via a kaolinite secondary in which the resonance has a known shift of -92.0 ppm (Harris et al., 2002). The corresponding ^31^P MAS NMR measurements were performed at 9.4 T using a Bruker Avance III spectrometer operating at Larmor frequency of 161.4 MHz. These experiments were performed using a Bruker 4 mm HX probe which enabled a MAS frequency of 12 kHz to be implemented. The ^31^P pulse time calibration was performed using ammonium dihydrogen phosphate (ADP) from which a π/2 pulse time of 2.5 μs was measured. All single pulse experiments were undertaken with a π/4 tip angle, a delay of 30 s, and a heteronuclear ^1^H/^31^P decoupling field of 100 kHz during data acquisition. All ^31^P chemical shifts were reported against the IUPAC recommended primary reference of H_3_PO_4_ (neat, δ = 0.0 ppm), via a ADP secondary in which the resonance has a known shift of 0.99 ppm (Harris et al., 2002). The distribution of Si speciation was analysed from quantitative measurement of the Q^n^ structures (i.e., Si(OSi)_n_ (OR)_4-n_ species (where R is Ca or H) derived from TEOS) and the T^n^ structures (i.e., C-Si(OSi)_n_(OR)_3-n_ species (where R is Ca or H) derived from the coupling agent GPTMS) from the ^29^Si MAS NMR data according to the equation (Connell et al., 2014):

$$\begin{array}{c} D_{c}(\text{\%})=100\text{\%}\times (\left[\frac{T^{1}+2T^{2}+3T^{3}}{3}\right]\\ +\left[\frac{Q^{1}+2Q^{2}+3Q^{3}+4Q^{4}}{4}\right]) \end{array}$$

All above-mentioned characterisations were performed on the 4 investigated hybrid compositions (Table 1) and compared with reference 100S-CL hybrid.

### Mechanical Testing on Hybrid Monoliths

Compression testing was carried out on cylindrical hybrid monoliths (H/Ø ≥ 1) using a Zwick/Roell Z010 machine equipped with a 10 kN load cell and set in displacement control. The bottom and top surfaces of each hybrid cylinder were flattened using SiC paper, before being placed between the compression platens. Uni-axial compression testing to failure was performed on all hybrid compositions at the displacement rate of 1 mm min^−1^ and 5 N pre-load (n ≥ 3): conventional stress (σ_c_) and conventional strain (ε_c_) were calculated as the ratio between the force and the nominal cross-section area and as the ratio between the actual displacement and the initial length of the sample, respectively; modulus of toughness (U_T_) was calculated by measuring the area underneath the compression stress-strain curve (where ε_c_ is unitless) using Origin Software (OriginLab Corporation, USA). Pure SiO_2_-CaO (60/40 mol%) glass, of the same composition as the inorganic part of the 60S40C-CL hybrid, identified as 60S40C, was also used as comparison for compression testing. The glass was made by conventional acid catalysed sol-gel, with the gel dried at 130 °C and stabilised at 700 °C (Lin et al., 2009; Lin et al., 2015).

With the same Zwick/Roell Z010 machine cyclic loading tests were performed on the 60S40C-CL hybrid monoliths (n = 3), applying 10 cycles following a 5 N pre-load at a displacement rate of 1 mm min^−1^ (in both loading and unloading) to 10%, 20% or 30% of the initial height, with a 3 min hold time (time interval between consecutive cycles) to let the sample recover the deformation before starting the next cycle. No dwell was used at the maximum strain.

### Dissolution in SBF and Bioactivity

SBF solution was chosen to evaluate dissolution and bioactivity of hybrid monoliths and was prepared following Kokubo’s protocol (Kokubo and Takadama, 2006). 150 mg of hybrid sample in powder form was immersed in 100 ml of SBF and placed in an orbital shaker at 37°C and 120 rpm shaking speed (Maçon et al., 2015). 1 ml of SBF supernatant was collected for Inductively Coupled Plasma Optical Emission Spectroscopy (ICP-OES) analysis at 0, 0.5, 1, 2, 4, 8, 24, 72, 168 and 336 h time points and replaced by 1 ml of fresh SBF. The pH of the SBF at each time point was also measured using pH meter with a thermal probe (Oakton pH 11 m with Oakton general-purpose glass pH probe, Ag/AgCl electrode, and Oakton ATC probe). The pH meter was calibrated at pH 4-7-10 before the measurements. For each sample, dissolution test was run in triplicates (n = 3). The powder samples were collected using filter paper (particle retention: 5–13 μm), washed with deionised water and then dried in a 40°C oven. The dry post-SBF immersion powder samples were ready for XRD and ATR-FTIR characterisations (with the same parameters reported above) to assess the possible apatite formation.

Si, Ca and P concentrations in the SBF samples collected at each time point for each sample were analysed by ICP-OES (Thermo Scientific iCAP 6,000 Series ICP). Before analysis, each solution was diluted by a factor of 10 with 2M nitric acid and standard solutions containing Si, Ca and P were prepared at 0, 2, 10 and 20 μg ml^−1^ for calibration.

### TRIS Degradation Study

Cylindrical hybrid monoliths were immersed in TRIS buffer solution (100 ml of TRIS solution for each sample) for a week to investigate the degradation by measuring the mass loss, i.e., weighing the samples before the test and after having been completely dried at room temperature in air after soaking. TRIS buffer solution was prepared following the protocol modified from Brauer *et al*. (Brauer et al., 2011), where 15.09 g of TRIS (hydroxymethyl) amino methane ((CH_2_OH)_3_CNH_2_) was dissolved in 1800 ml of deionised water, adding 44 ml 1M HCl, left at 37°C overnight, filled to a total volume of 2000 ml with deionised water and then adjusted the pH to 7.40 at 37°C with 1M HCl. The variation of the I/O ratios of the hybrids after degradation in TRIS were determined using DSC/TGA (with the same setup described above) on the samples that were completely dried after the soaking and manually ground with a pestle and mortar.

### Cell Culture Study on Monoliths

#### In vitro Cell Culture

MC3T3-E1 preosteoblast cell line (ATCC, UK) were expanded in basal α-MEM monolayer culture supplemented with 10% (v/v) foetal calf serum (FCS), 100 μg ml^−1^ streptomycin and 100 U ml^−1^ penicillin. Cultures were maintained in at 37°C in a humidified atmosphere of 21% O_2_ and 5% CO_2_. At confluency, cells were passaged using 500 μg ml^−1^ trypsin-EDTA (ethylene diamine tetra-acetic acid).

#### Cytotoxicity Testing

Potential *in vitro* cytotoxicity of hybrids on the MC3T3-E1 cells was assessed according to ISO 10993-5 (Standardization, I. O. F., 2009) and ISO 10993-12 (Standardization, I. O. F., 2012). Powders were sterilised in 70% ethanol for 1 min, rinsed with PBS (Phosphate Buffered Saline) and immersed (0.2 g ml^−1^) in serum free α-MEM at 37°C for 72 h. The conditioned media was then syringe filtered (particle retention of 0.2 μm) and collected for the cell culture. The conditioned media were diluted to 25%, 50%, 75% and 100% and supplemented with 10% (v/v) FCS for use in the cell viability assays. Medical grade polyethylene (PE) was used as negative control (non-cytotoxic) and polyurethane (PU) containing 0.1% (w/w) zinc diethyldithiocarbamate (ZDEC) was used as positive control (reproducibly cytotoxic).

96-well plates were used, where each well contained 1 × 10^4^ MC3T3-E1 cells, which were grown in basal α-MEM for 24 h until a sub-confluent monolayer was formed. The basal α-MEM was replaced with the dissolution products of PCL hybrid materials or with fresh basal α-MEM (control) or the dissolution products of the negative and positive controls (100 μl well^−1^) for a further 24 h of culture. The media was replaced with solutions of MTT in serum-free α-MEM at a concentration of 1 mg ml^−1^ (50 μl well^−1^). After 2 h of incubation, the MTT solution was removed and 100 μl of isopropanol was added to dissolve the formazan derivatives (formazan crystals). A microplate reader (SpectraMax M5) was used to measure the optical density of the resultant solution at 570 nm spectrophotometrically.

#### Cell Culture on Hybrid Discs

For cell attachment studies, hybrid discs (approximately 5 × 5 × 1 mm^3^) were sterilised with 70% ethanol for 1 min and rinsed with PBS. Then each sample disc was immersed in fresh serum free α-MEM for 30 min before cell seeding. MC3T3-E1 cells were suspended in basal α-MEM at a concentration of 1×10^6^ cells ml^−1^. 10 μl of cell suspension was seeded onto each hybrid disc and incubated for 2 h at 37°C, in a humidified atmosphere of 5% CO_2_ and 21% O_2_. Then fresh basal α-MEM was added to submerge each cell-seeded disc and samples were cultured for further 24 h.

#### Immunohistochemistry Staining and Confocal Microscopy

4% paraformaldehyde (PFA) was used to fix the membranes of attached cells. Buffered 0.5% Triton X-100 in PBS (300 mM sucrose, 50mM NaCl, 3 mM MgCl_2_, 20 mM HEPES and pH 7.2) was used for permeabilisation of the cell membranes and 10 mg ml^–1^ Bovine Serum Albumin (BSA) in PBS was used for blocking nonspecific epitopes. Samples were then incubated with anti-Tubulin antiserum (1:200 dilution in 10 mg ml^−1^ BSA/PBS, rabbit polyclonal, IgG, Abcam, Cambridge, UK) at 4°C for 1 h. This was followed by 1 h incubation with Alexa Fluor® 488-conjugated secondary antibody (1:1000 dilution in 10 mg ml^−1^ BSA/PBS, goat IgG, Abcam, Cambridge, UK). Negative controls (omission of the primary antisera with the presence of secondary antibodies) were performed in all immunohistochemistry procedures. No staining was observed in the samples used as negative controls.

F-actin was labelled using CytoPainter F-actin staining kit (Abcam, Cambridge, UK) following the manufacture’s instruction. Briefly, Alexa Fluor® 568-conjugated phalloidin (1: 1000 dilution in labelling buffer) was added simultaneously with the secondary antibody during the incubation period. All samples were counter-stained with DAPI (0.1 μg ml^−1^ in PBS) nuclei stain. Then the samples were imaged under confocal microscopy (Leica SP5 MP laser scanning confocal microscope and software, Leica Microsystems, Wetzlar, Germany).

## Results

### Characterisation of the Hybrid Network

**Figure 1:**
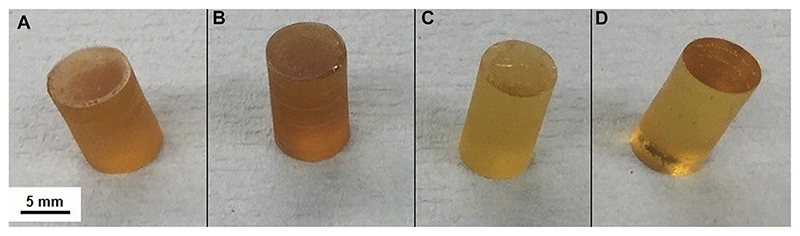
Photographs of representative SiO_2_-CaO-P_2_O_5_/PTHF/PCL-diCOOH hybrid monoliths: **(A)** 60S40C-CL, **(B)** 60S36C4P-CL, **(C)** 60S32C8P-CL, **(D)** 60S28C12P-CL.

SiO_2_-CaO-P_2_O_5_/PTHF/PCL-diCOOH hybrid monoliths, with inorganic component containing Ca and P, as per Table 1, were successfully synthesised by modification of the sol-gel synthesis involving THF polymerisation (Tallia et al., 2018) with the addition of CME and TEP. Figure 1 shows all hybrids could form crack-free homogeneous cylinders (Ø ≈ 8.3–9.1 mm; H ≈ 10–14 mm), proving that the shrinkage during drying occurred along the three spatial dimensions, with a yellow-orange coloration typical of other silica-based hybrid materials (Li et al., 2015; Chung et al., 2016; Tallia et al., 2018). All hybrid compositions were made starting from the same TEOS/PCL ratio of 80/20 wt% (i.e., 100S-CL) (Tallia et al., 2018), to which the calculated volume of CME and TEP needed to achieve the desired inorganic composition was added. Hence, the addition of Ca and P ions to the inorganic part (analogous to a bioactive glass of 60 mol% of SiO_2_ and a different proportion of CaO and P_2_O_5_ from 40/0 to 28/12 mol %) led to an increase of the final inorganic/organic ratio of the resulting hybrid samples: as confirmed by DSC/TGA analysis (Table 1, Supplementary Figure S1). The four hybrid compositions were characterised by an inorganic content of 31.8 ± 1.7 wt%, approximately 7 wt% higher than the corresponding Ca-free and P-free hybrid composition made from the same TEOS/PCL-diCOOH = 80/20 wt% (i.e., 100S-CL) (Tallia et al., 2018). As expected, the substitution of 40 mol% of silica with modifying cations, while maintaining a TEOS/PCL-diCOOH of 80/20 wt%, did increase the total inorganic content. However, the fact that the four hybrid compositions possessed similar I/O ratios independent of the CaO/P_2_O_5_ ratio showed that CME and TEP did not affect polymerisation of THF. DSC traces for all four compositions displayed a sharp peak around 210°C and broader band between 300 and 400°C compatible with the burning-out of the two organic phases (Supplementary Figure S1). From analysis of commercially available polymers, it was found that PTHF burns around 200–280°C, whereas PCL burns at a higher temperature between 300 and 400°C. Hence, even if it is likely that there was an overlap in the burn-out of the two polymers, the first more prominent peak in the hybrid DSC traces was attributed predominantly to PTHF, whereas the second broader peak was attributed mainly to PCL-diCOOH.

**Figure 2:**
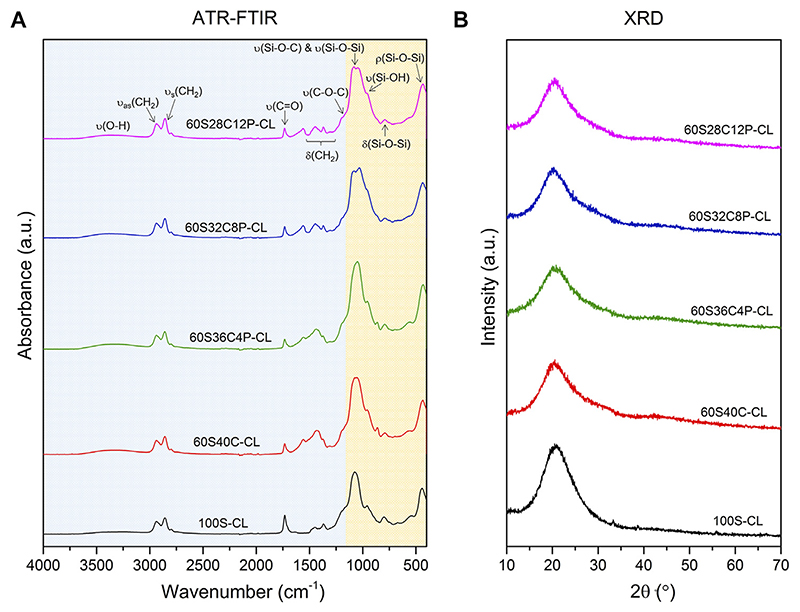
ATR-FTIR spectra **(A)** and XRD patterns **(B)** of the SiO_2_-CaO-P_2_O_5_/PTHF/PCL-diCOOH hybrid compositions: in **(A)** the region highlighted in orange (~450–1,200 cm^−1^) represents the contribution of the inorganic component, while the region highlighted in blue (~1,200–4,000 cm^−1^) represents the contribution of the organic components (i.e., PTHF and PCL).

The hybrid networks were characterised with ATR-FTIR, XRD and solid-state NMR. In all the ATR-FTIR spectra (Figure 2A), the contribution of both inorganic and organic components is visible (Tallia et al., 2018). Bands from ~450 cm^−1^ to ~1,200 cm^−1^ are mainly attributed to the inorganic component (Zhang et al., 2007), with bands present similar to those found in FTIR spectra of bioactive glasses, whereas the bands visible at higher wavenumbers are mainly due to the organic contribution to the hybrid system (Elzein et al., 2004; Sahoo et al., 2010; Zhang et al., 2012). The band at 470 cm^–1^ was attributed to the rocking vibration of the bridging oxygen atoms perpendicularly to the Si-O-Si plane, and the band at 800 cm^−1^ is the Si-O-Si bending (Chrissanthopoulos et al., 2008). The band at around 950 cm^−1^ was assigned to the stretching vibration of two non-bridging oxygen atoms in the Q^2^ tetrahedral unit of Si-O-Si (Domine and Piriou, 1983; Pryce and Hench, 2004). A weak Si-OH band at 960 cm^−1^ overlapped the Q^2^ tetrahedral Si-O-Si band (Stolen and Walrafen, 1976; Melinte et al., 2011). A shoulder at 1,250 cm^−1^ was assigned to the C-O-C stretching, from the organic part of the hybrid. The band at ~1725–1730 cm^−1^ was ascribed to the stretching of the carbonyl (C=O) from PCL-diCOOH (Elzein et al., 2004). The bands between 1,550 and 1,560 cm^−1^ and 1,430–1,450 cm^−1^ were assigned to the Ca^2+^ mono- and bidentate coordination mode (Mizuguchi et al., 1997) by the COO^−^ groups of PCL-diCOOH carboxylate respectively, whereas the band at 1,365 cm^−1^ was assigned to free COO^−^ groups (Nara et al., 1994; Nara et al., 2013). The bands at ~2,940 cm^−1^ and 2,858 cm^−1^ were attributed to asymmetric CH_2_ stretching and symmetric CH_2_ stretching of the organic part respectively (Elzein et al., 2004; Zhang et al., 2012). The low broad band at 3,250 cm^−1^ indicate the residual hydroxyl (OH) stretching vibration from the -OH groups present in both organic and inorganic component of the hybrid system after the drying process (Zhang et al., 2012; Tallia et al., 2018). The remaining bands between 1,250 and 1,550 cm^−1^ were assigned to the bending (i.e., scissoring) of CH_2_ groups from both PCL and PTHF (Sahoo et al., 2010; Zhang et al., 2012).

All XRD patterns of the hybrids exhibited an amorphous halo centred around 2θ = 20–23° (Figure 2B), which demonstrated that no crystalline phases formed in the hybrid network. These results, combined with the visible translucency of the samples, suggest that the additional inorganic ions did not affect the true interpenetration of the organic and inorganic co-network observed in the Ca-free hybrid under TEM investigation.

**Figure 3:**
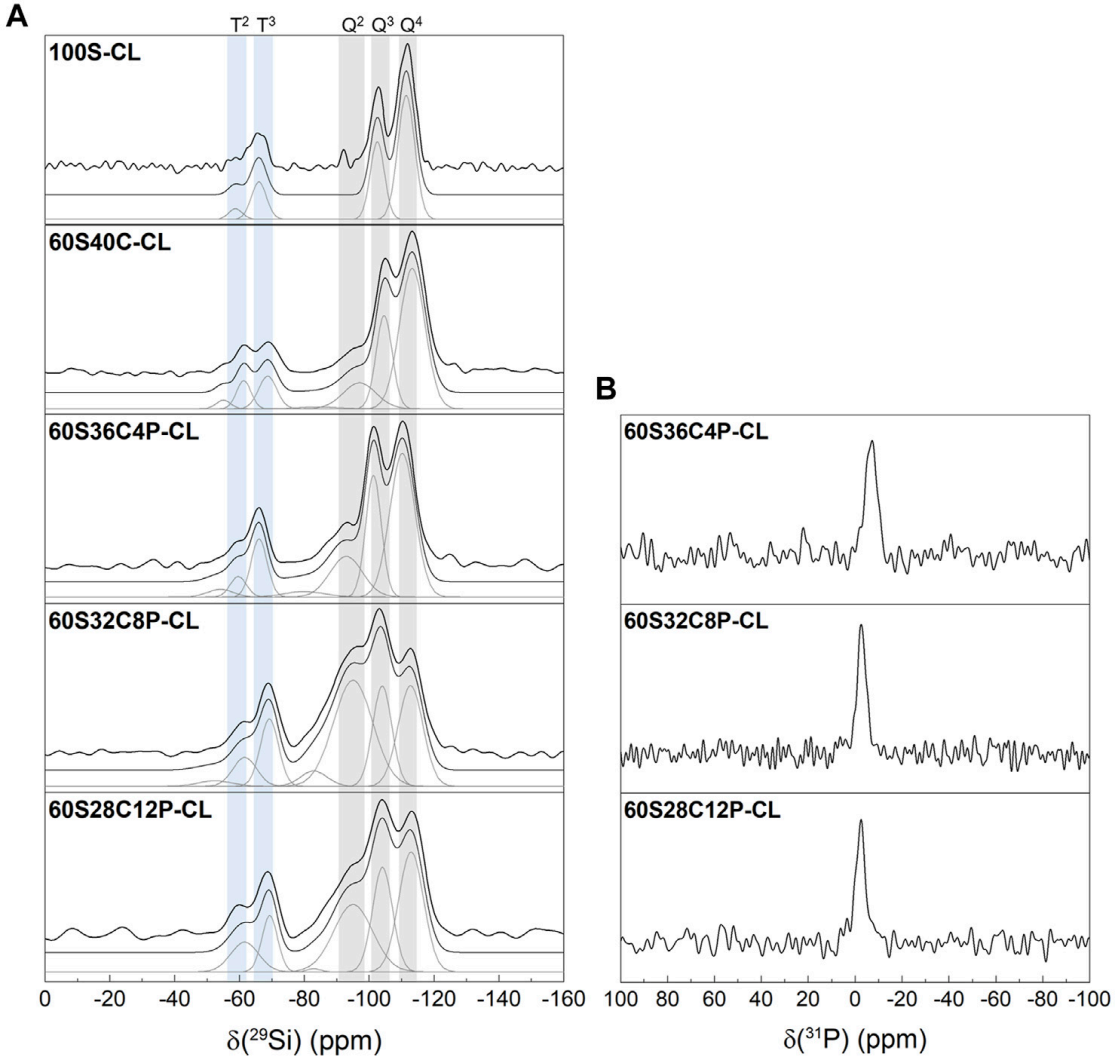
Single pulse solid-state MAS NMR of the SiO_2_-CaO-P_2_O_5_/PTHF/PCL-diCOOH hybrids: **(A)** ^29^Si MAS NMR with shaded regions highlighting peaks attributed to Q and T species following the peak fitting to calculate the abundance of each silicon species; **(B)** ^31^P MAS NMR.

Solid state ^29^Si NMR of the SiO_2_-CaO-P_2_O_5_/PTHF/PCL-diCOOH hybrids and of reference 100S-CL hybrid was carried out to identify the molecular conditions of silane groups from TEOS (Q^n^ species) and GPTMS (T^n^ species), and to obtain information on the silica network (Figure 3A). Table 2 shows the relative quantities of each Q^n^ and T^n^ species and the corresponding chemical shift and calculated D_c_. The reference 100S-CL hybrid showed the highest degree of condensation, with T^1^, Q^1^ and Q^2^ species not even detected: this agrees with theoretical expectations, considering the absence of network modifiers that could disrupt the silica network. The calculated value of D_c_ (~92%) was in the same range of those measured on sol-gel hybrid materials prepared via sol-gel route using TEOS as single organic component and GPTMS as coupling agent with different organic components, such as γ-PGA (Poologasundarampillai et al., 2014), chitosan (Connell et al., 2014) and gelatin (Mahony et al., 2010). Introducing Ca (without phosphate) decreased D_c_ to 85%, as expected, as calcium became a network modifier in the silicate network, increasing the relative proportions of Q^2^ and T^2^ units. Unexpectedly, when 4 mol% P_2_O_5_ was introduced at the expense of Ca, D_c_ decreased to 82% and as P_2_O_5_ increased to 8 mol% P_2_O_5_, D_c_ decreased further to 72%. When P_2_O_5_ increased to 12 mol%, instead, D_c_ increased to 77%.

Two possible hypotheses can explain this. One is the interaction of calcium ions within the organic network, in addition to their role in the silicate network. Ca^2+^ can establish ionic bonds with COO^−^ terminal groups in PCL-diCOOH, forming carboxylates (Valliant et al., 2013). Indeed, as GPTMS reacted with THF, causing its polymerisation, the system was left with an excess of COO^−^ groups, which were then available for Ca^2+^ chelation. A second hypothesis is that the role of the phosphate was similar to that reported previously for solgel glasses, wherein the P_2_O_5_ was present as orthophosphate for 4 mol% P_2_O_5_ and 8 mol% P_2_O_5_ and then, as phosphate content increased, some of the phosphate formed a phosphate network. However, the ^31^P NMR does not show evidence of polyphosphate (which would normally give a signal around -25 ppm but none is detected here), suggesting that only orthophosphate was present in all the P-containing hybrids (Figure 3B) (Ren et al., 2017). So, when present, the phosphate is likely charge balancing Ca^2+^ without forming Si-O-P bonds (Ting et al., 2017). Therefore, the unexpected D_c_ trend is likely due to calcium interacting with PCL-diCOOH, when the calcium content was high, in preference to incorporating in the silica network. Conversely, when phosphate was present, orthophosphate units interfered with the Ca^2+^-COO^-^ ionic interaction, pushing calcium to incorporate in silica network rather than in the organic component. According to this hypothesis, the Dc values suggest that, among the three P-containing compositions, 60S32C8P-CL is the hybrid where the highest proportion of calcium, relatively to silica, is incorporated in silica network.

### Mechanical Properties

**Figure 4:**
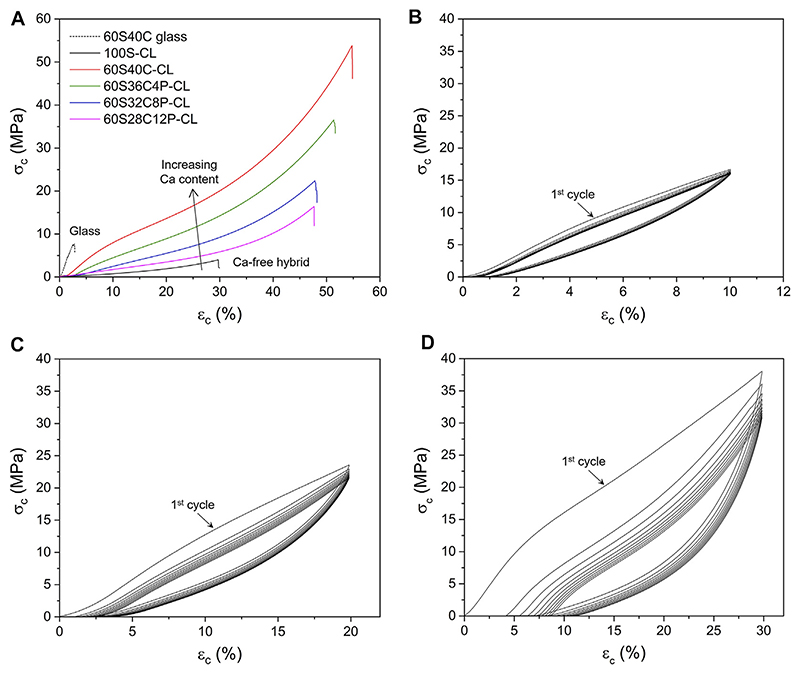
Compression testing of SiO_2_-CaO-P_2_O_5_/PTHF/PCL-diCOOH hybrid monoliths: **(A)** Example conventional stress-strain curves to failure, comparing the four Ca-containing hybrid samples with pure 60S40C (60 mol% SiO_2_, 40 mol% CaO) glass and Ca-free hybrid **(B–C–D)** Example cyclic testing curves for 60S40C-CL (SiO_2_-CaO-/PTHF/PCL-diCOOH) hybrid monoliths compressed up to 10% **(B)**, 20% **(C)** and 30% **(D)** of their initial height.

While the properties of the 100S-CL hybrid were deemed suitable for articular cartilage regeneration (Tallia et al., 2018), increased stiffness is thought to be needed for bone regeneration, while maintaining high toughness and introducing bioactivity through the introduction of calcium and phosphate (Rho et al., 1998; Osterhoff et al., 2016). Hybrid monoliths of the four compositions (Table 1) were subjected to compression testing and compared to reference 100S-CL hybrid and to 60S40C glass (no organic component). Example stress-strain curves are reported in Figure 4A and stress, strain and modulus of toughness values at failure are reported in Table 3. The stress-strain curves (Figure 4A) of all the calcium-containing hybrids expressed a non-linear relationship, with a tendency to be stiffer at higher strain, suggesting a viscoelastic property of the Ca-containing hybrids. The addition of Ca and P significantly improved the mechanical properties of the 100S-CL hybrid samples, since all the Ca-containing hybrid compositions had much greater σ_c_, ε_c_ and U_T_ at failure compared to Ca-free hybrid sample (Table 3). All four Ca-containing hybrids showed a modulus of toughness of 1-2 orders of magnitude higher than 100S-CL. Among the Ca-containing compositions, the P-free 60S40C-CL exhibited the best mechanical properties in compression (Figure 4A): the conventional compression stress and strain at failure were 3.3 ± 0.9 MPa and 27.9 ± 3.3%, respectively, for 100S-CL hybrid cylinders; while 60S40C-CL had stress and strain at failure of 63.7 ± 20.9 MPa and 55.9 ± 5.1%, respectively. Hence, the presence of calcium rather than phosphorous seems to be the key factor in increasing strength and toughness of the SiO_2_/PTHF/PCL-diCOOH hybrid system. The increase in properties was so great that the few wt% mismatch in I/O content will not have had an impact. A possible explanation might be related to the ionic interaction between COO^−^ in the PCL component of the hybrid and the Ca^2+^ ions in the inorganic part (as indicated by the NMR results): this ionic crosslinking represents additional bonding between the inorganic and organic components of the hybrid co-network, contributing to increase toughness and strength of the material. Further investigation is needed to confirm this hypothesis, but it would also be consistent with the observation that the higher the phosphate content (hence, the lower the calcium content), the less stiff the hybrid was (Figure 4A; Table 3). As demonstrated by NMR analysis, phosphate is present as orthophosphate ions, which are then incorporated into a silica network by charge balancing with calcium ions, reducing the number of Ca^2+^ ions available for ionic bonding with PCL-diCOOH.

Since the Ca-containing SiO_2_/PTHF/PCL-diCOOH hybrids made in this work were designed to be used for bone regeneration, it is important to investigate their behaviour under cyclic loading. The 60S40C-CL hybrid was chosen for cyclic loading tests because it exhibited the best mechanical property among all the hybrid compositions in compression to failure (Figure 4A). Three strain intervals were investigated: up to 10–20–30% of the initial height of the samples, which were all below the failure average strain (~56%). When 60S40C-CL samples were repeatedly compressed to 10% of the initial height for 10 cycles, all cycles almost overlapped (Figure 4B). The hold time between each cycle was set to 3 min and that was enough for the hybrid to recover the 10% deformation, which is why all the cycles were almost overlapping. When 60S40C-CL samples were compressed to 20% or 30% of the initial height for 10 loading-unloading cycles (Figures 4C,D respectively), the first cycle showed a different stress-strain behaviour to the following 9 cycles. Moreover, differently from the test to 10% strain, consecutive cycles displayed a shift of the starting point of the curve towards values >0, showing that 3 min hold time between each cycle was not enough for the hybrid to fully recover the deformation (20% or 30%), with the effect being more evident the higher the maximum strain. At 20% and 30% strain, a stress-softening effect was also observed, where the stress at a given strain dropped between the successive loading cycles. The stress drop was significant between the first and second cycles, then it started to decrease, becoming negligible after 5 or 6 cycles. This stress-softening effect was also observed previously on Ca-free hybrids, where the possible explanation was ascribed to the Mullins effect, which is typical of elastomers (Tallia et al., 2018). According to the Mullins effect, under mechanical stress the organic chains can slip over one another and rearrange to accompany the stress, causing temporary elimination of the entanglements in the organic component of the hybrid network and creating an internal stress in the hybrid material (Cantournet et al., 2009). After the external stress is removed, the accumulated internal stress causes the hybrid to return to the original form and the entanglements can also recover slowly. When the hybrid was compressed to 10% deformation, most of the entanglements were recovered within 3 min, which explains why the stress-softening effect was negligible in 10% deformation cyclic loading (Figure 4B). When the hybrid was compressed by up to 20% (Figure 4C) or 30% (Figure 4D) deformation, some entanglements were not able to be recovered and other entanglements might take longer than 3 min to fully recover. Most of the unrecovered entanglements were removed in the first few cycles, causing the significant reduction in stress at a given strain between the first and second cycles, and the drop in stress became negligible after about 5 or 6 cycles. These results suggest that a mechanical preconditioning of the samples (i.e., cyclic loading for ≥6 cycles) would be recommended before their use *in vivo*. It is important to underline that 10% strain (i.e., range in which 60S40C-CL hybrid did not show significant stress-softening) is already in excess of the compressive strain reported for bone application (i.e., up to 7% for trabecular bone (Burstein et al., 1976)).

Small hysteresis (more evident the higher the maximum strain) between loading and unloading was observed for all 10 cycles in all the cyclic loading tests, indicating that 60S40C-CL hybrid was viscoelastic (i.e., it showed a time-dependent elastic behaviour). This means that, in accordance with the Mullins effect previously described, the temporarily eliminated entanglements in the organic chains were not instantly recovered when the load was removed, but required some time to recover completely. Also bone is a viscoelastic material, where its time-dependant deformation under loading arises from multiple factors related to its structure on multiple length scales (Mano, 2005; Wu et al., 2012). After the first preconditioning cycle, negligible hysteresis was instead observed for 100S-CL scaffolds under cyclic loading (Tallia et al., 2018), demonstrating further the suitability for trabecular bone application of the hybrid system when Ca is added.

### Dissolution in SBF

**Figure 5:**
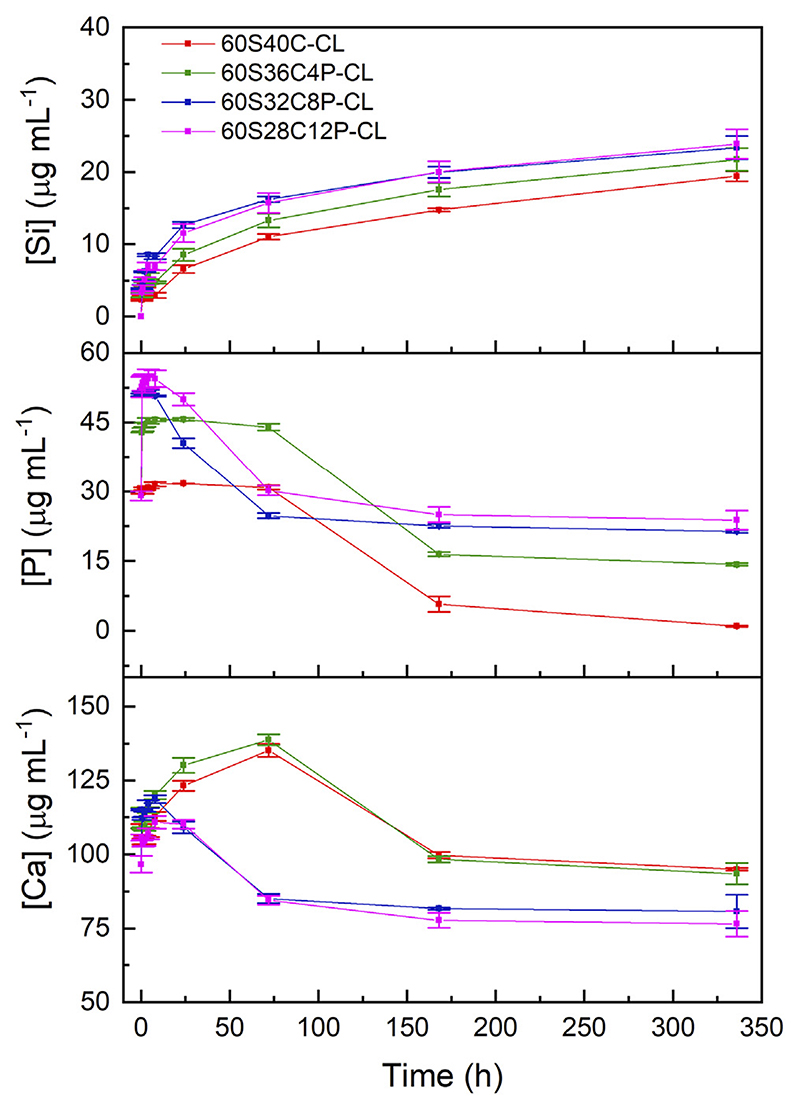
ICP-OES of SBF after immersion of SiO_2_-CaO-P_2_O_5_/PTHF/PCL-diCOOH hybrid powders. Error bars are standard deviations from the mean values for n = 3.

The amounts of dissolution products from each Ca-containing hybrid in SBF were measured by ICP-OES. The pH of the SBF increased in the first 24 h after sample immersion, after which it plateaued; all hybrids displayed very similar pH buffering ability, with pH within the range 7.40 ± 0.25, which is compatible with cell survival. The phosphate was released from all the P-containing hybrids rapidly within the first 30 min of SBF immersion (Figure 5), which was expected as the phosphate was present as orthophosphate units. After 30 min of SBF immersion: the [P] in SBF containing 60S36C4P-CL increased from 30 μg ml^−1^ (initial [P] of SBF) to around 45 μg ml^−1^; for SBF containing 60S32C8P-CL and 60S28C12P-CL the [P] increased to around 55 μg ml^-1^. Between 8 and 144 h of SBF immersion, the phosphate concentration in SBF from 60S32C8P-CL and 60S28C12P-CL reduced significantly; but the phosphate level in SBF containing 60S40C-CL and 60S36C4P-CL stayed constant and only started dropping after 72 h of SBF immersion.

The ICP profile of calcium (Figure 5) shows rapid release of calcium from all the hybrids within the first 8 h of SBF dissolution. The [Ca] in SBF at time point 0 was around 100 μg ml^−1^; after 8 h of SBF immersion, the [Ca] increased in SBF containing 60S40C-CL to 112 μg ml^−1^; to 120 μg ml^−1^ for SBF containing 60S36C4P-CL; to 118 μg ml^−1^ for SBF containing 60S32C8P-CL and to around 110 μg ml^−1^ for SBF containing 60S28C12P-CL. [Ca] in the SBF containing 60S32C8P-CL and 60S28C12P-CL dropped after 8 h of immersion, whereas SBF containing 60S40C-CL and 60S36C4P-CL continued to release calcium into until 72 h of soaking in SBF, after which [Ca] decreased.

The drop in the concentrations of phosphate and calcium indicates the deposition of phosphate and calcium to form amorphous calcium phosphate on the surface of the hybrid. There are two possible explanations for the calcium and phosphate ICP profiles: 1) all the four hybrids released calcium ions consistently, but the calcium and phosphate deposited faster onto 60S32C8P-CL and 60S28C12P-CL when compared to 60S40C-CL and 60S36C4P-CL; 2) there was a consistent release of calcium ions from 60S40C-CL and 60S36C4P-CL until 72 h from soaking in SBF, but not from 60S32C8P-CL and 60S28C12P-CL. The consistent release of calcium from 60S40C-CL and 60S36C4P-CL could possibly be due to of the presence of PCL and PTHF in the hybrid network.

An unexpected result was obtained for Si ICP profile, which gives information about the degradation rate of the inorganic part of the hybrid. Figure 5 shows that the higher the calcium content in the hybrid (i.e., the lower the phosphate content) the slower the Si release, which suggests that the glass component in the hybrid degraded slower when the calcium content was higher. It is completely opposite to the results from biodegradation of SiO_2_-CaO-P_2_O_5_ sol-gel glasses obtained previously (Ting et al., 2017), but it is consistent with the D_c_ calculated from ^29^Si-NMR (Table 2). This supports the hypothesis that a portion of the Ca^2+^ (and consequently of the charge-balancing phosphate ions) interacted with COO^−^ from PCL-diCOOH instead of being all incorporated in the silicate network. The ionic interaction between Ca^2+^ and PCL-diCOOH chains also explains the consistent release of calcium from 60S40C-CL and 60S36C4P-CL. However, further studies are needed to confirm this. Previous ICP-OES studies showed that the Si release from Ca-free hybrids in PBS was negligible (i.e., few μg ml^−1^ after soaking for 7 days) (Tallia et al., 2018), hence the addition of Ca and P in the glass network increased the dissolution rate of the inorganic component.

**Figure 6:**
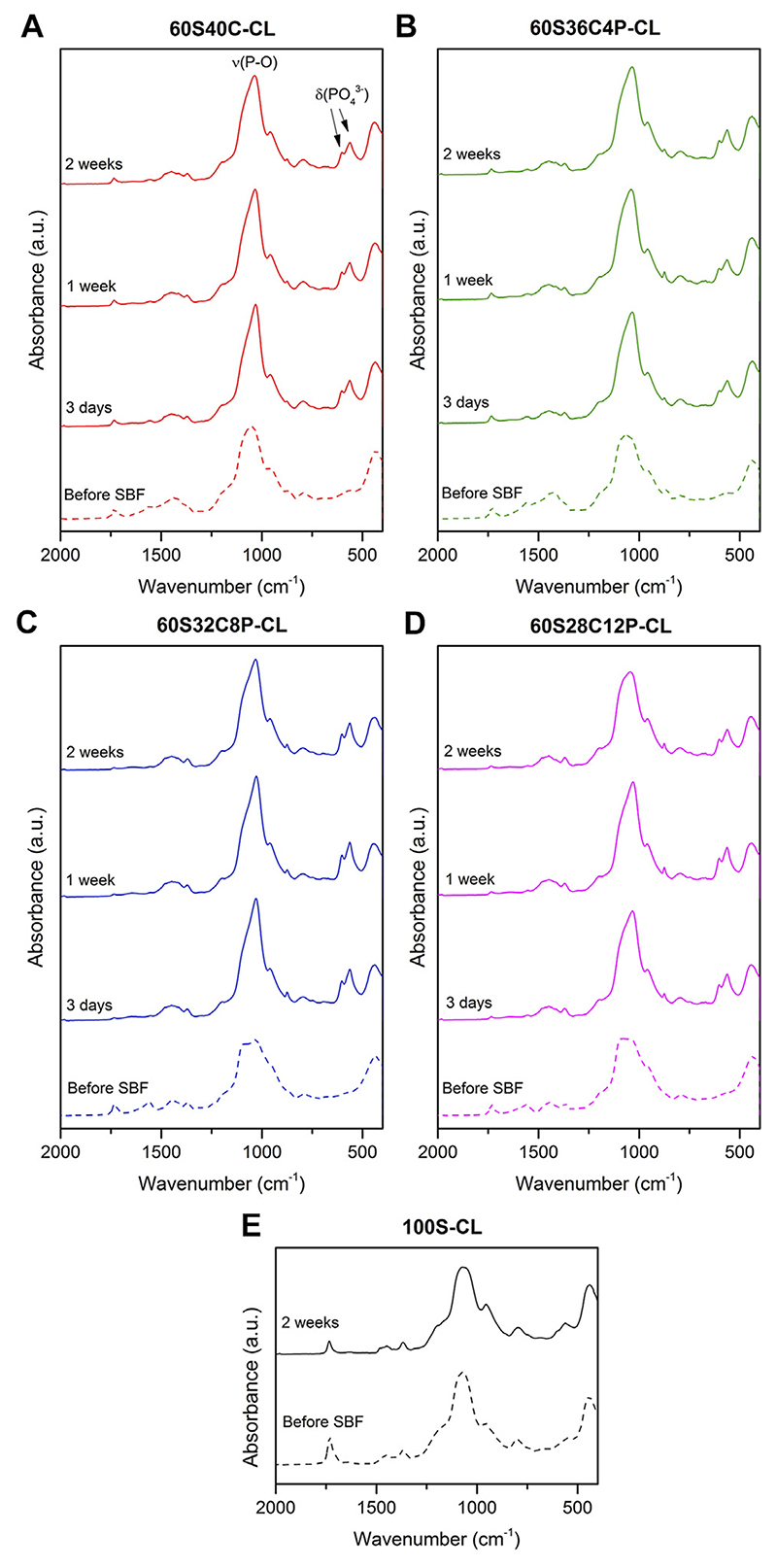
**(A–D)** ATR-FTIR spectra of the four Ca-containing hybrid compositions before and after soaking in SBF for 3, 7 and14 days: **(A)** 60S40C-CL, **(B)** 60S36C4P-CL, **(C)** 60S32C8P-CL, **(D)** 60S28C12P-CL; **(E)** ATR-FTIR spectra of the reference Ca-free hybrid before and after 14 days in SBF.

The possible calcium phosphate/apatite formation at 3-7-14 days of SBF soaking was evaluated via ATR-FTIR and XRD analysis. The double bands at 546 cm^−1^ and 585 cm^−1^ visible in the ATR-FTIR spectra of all the soaked hybrids (Figures 6A–D) are attributed to the P-O asymmetric bending motion in the orthophosphates, and the other band at 1,010 cm^–1^ (Figures 6A–D) corresponds to P-O stretching vibration of PO_4_^3-^. These three bands indicate the presence of apatite in the hybrids. The C-O stretching single band at around 862 cm^−1^ indicates the presence of CO_2_^3-^ groups, which suggests that the formed apatite was hydroxycarbonate apatite (HCA) (Ting et al., 2017). From the ATR-FTIR spectra, it can be concluded that all the Ca-containing hybrids (60S40C-CL, 60S36C4P-CL, 60S32C8P-CL, 60S28C12P-CL) induced HCA formation on the surface just after 3 days of SBF immersion. There was no difference in the HCA formation ability among hybrids with different glass compositions incorporated as inorganic component. No apatite formation was detected on calcium free hybrids even after 2 weeks in SBF (Figure 6E).

**Figure 7:**
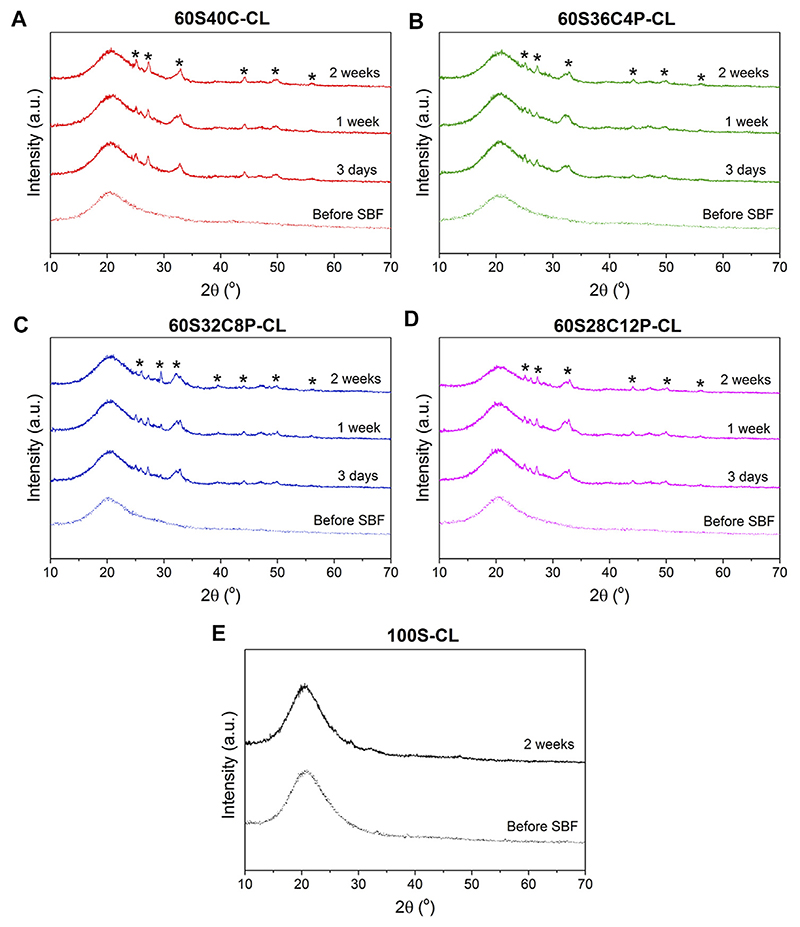
**(A-D)** XRD patterns of the four Ca-containing hybrid compositions before and after soaking in SBF for 3-7-14 days: **(A)** 60S40C-CL, **(B)** 60S36C4P-CL, **(C)** 60S32C8P-CL, **(D)** 60S28C12P-CL; **(E)** XRD patterns of the reference Ca-free hybrid before and after 14 days in SBF. * notates peaks matching ICDD card number 00-019–0,272 carbonate-hydroxylapatite, syn phase (PDF index name calcium carbonate phosphate hydroxide) (Legeros et al., 1967).

Figure 7 shows XRD patterns for hybrids before and after SBF dissolution, confirming what was observed from ATR-FTIR analysis. Peaks at around 2θ = 25°-32°-46°-49° were visible from the first investigated time-point (3 days) for all four Ca-containing hybrids (Figures 7A–D). This was attributed to the HCA formed during SBF immersion. However, the HCA peaks detected in hybrids were slightly different to those of bioactive glasses (Ting et al., 2017; Araujo et al., 2021). It indicates that the HCA crystals formed on hybrids have slightly different preferred growth orientation to the HCA crystal formed on bioactive glasses. The XRD data also confirmed that no apatite was formed on reference 100S-CL hybrid after 2 weeks of SBF immersion (Figure 7E).

ATR-FTIR and XRD data suggest that: 1) all the Ca-containing hybrids (60S40C-CL, 60S36C4P-CL, 60S32C8P-CL, 60S28C12P-CL) were able to induce HCA formation just after 3 days of SBF dissolution; 2) adding/increasing phosphate content within the tested range did not affect HCA forming ability as no significant difference among hybrids were detected; 3) Ca-free hybrids were not able to induce apatite formation.

### TRIS Degradation Study

The Ca-containing hybrids were also soaked in TRIS buffer for 1 week to determine the weight loss rate, without any apatite formation, and to compare I/O ratio before and after soaking in TRIS buffer. Supplementary Figure S2 shows photographs of cylindrical hybrid monoliths after 1 week of TRIS dissolution. All the cylindrical monoliths maintained their shape and were integral when in solution, during water uptake; but after drying, many cracks appeared. Attraction of the polar water molecules to the hydrophilic silica component in the hybrid contrasts to the hydrophobic nature of both PCL (Shih et al., 2014) and PTHF (Pol et al., 1996a; Pol et al., 1996b), which caused cracking as the water tried to escape the hybrids during drying. Kamitakahara *et al*. previously described the penetration of water in the interior of TiO_2_/PTHF hybrids, despite the hydrophobicity of the polymer (Kamitakahara et al., 2003). The amount of adsorbed water by the hybrids was low, in that no changes in volume were noticed. Hence, it is believed that the polymers tend to rearrange in a different chain organisation that limits the contact with water, to which they have poor affinity. The water uptake and the following rearrangement may cause some internal tensions (at the molecular level) between the hydrophobic organic components and the hydrophilic inorganic phase. However, the water uptake was limited and the hybrids achieved an equilibrium when in solution, where their integrity was preserved. Since it is due to the affinity with the silicate, the water uptake and the consequent internal stress had a higher impact on samples containing high inorganic fraction.

During drying after soaking, water was removed and everything tended to return to the initial state. At that point, a further distinction could be observed depending on the amount of polymer, the rearrangement of which played a crucial role: for inorganic content below ~30 wt% (e.g., 100S-CL), limited internal stresses were involved and the percentage of organic was enough to accommodate variations over drying; this would explain the fact that 100S-CL did not show any macroscopic change when soaked in a neutral solution (i.e., PBS) (Tallia et al., 2018). The opposite was true for inorganic content around 30 wt%, wherein the lower organic content was not able to compensate the fast release of the higher stresses (because of the higher percentage of hydrophilic phase), resulting in the formation of cracks.

Values of weight loss and variation in the I/O ratio reported in Table 4 demonstrated that there was not a strong correlation between the weight loss and the inorganic compositions. The inorganic content before and after TRIS buffer dissolution was <1.5 wt% difference for 60S40C-CL, 60S36C4P-CL and 60S32C8P-CL, meaning that the hybrids lost organic and inorganic at consistent and similar rate in TRIS buffer. This was due to the combined effect of water entering the hybrid during the soaking in TRIS, which caused both hydrolysis of PCL-diCOOH (Woodruff and Hutmacher, 2010) and degradation of the glass network. A slightly higher difference (4 wt% inorganic increase, with 10.8 wt% mass loss) was detected for 60S28C12P-CL. This was similar to the 3.2 wt% increase of inorganic content and 12 wt% mass loss of calcium and phosphate-free 100S-CL 3D printed scaffolds reported previously (Tallia et al., 2018) after 1 week in PBS, from which it was concluded that any mass loss in solution was mainly PCL degradation.

### Cell Culture Study

**Figure 8:**
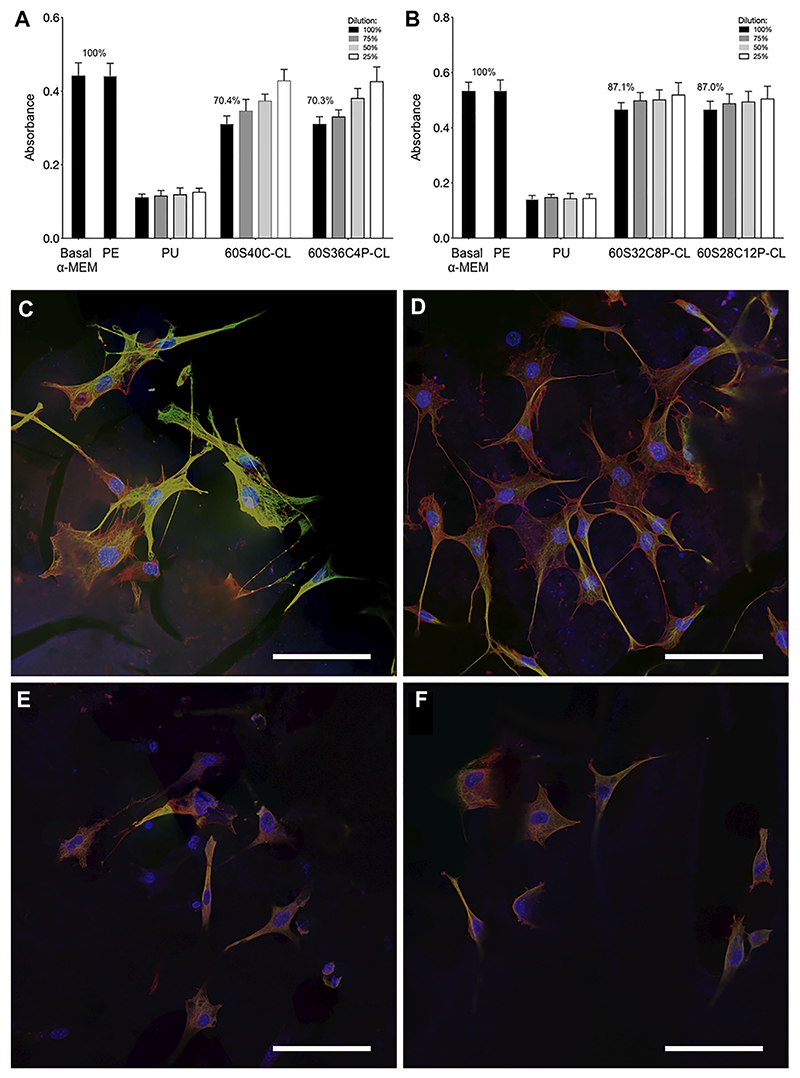
Preosteoblast cell cultures following exposure to dissolution products of the hybrids and culture on the surface of monoliths: **(A,B)** Metabolic activity of the preosteoblast cells after 24 h of incubation with the dissolution products of each of the four Ca-containing hybrids **(C–F)** confocal microscopy images of the preosteoblast cells cultured on the surface of **(C)** 60S40C-CL, **(D)** 60S36C4P-CL, **(E)** 60S32C8P-CL, **(F)** 60S28C12P-CL. Scale bars = 100 μm in **(C–F)**. Data points are mean ± S.D. Statistical analysis was performed using Kruskal–Wallis test with Dunn’s post test in Prism 7. Results were deemed significant if the probability of occurrence by random chance alone was less than 5% (i.e., *p* < 0.05).

Cytotoxicity and cell attachment tests were performed on the hybrids using the MC3T3-E1 preosteoblast cell line. For a material to pass ISO 10993-5 for toxicity, the viability of cells (in the MTT assay) when in contact with the dissolution products (from the testing material) needs to be above 70% in comparison to basal and negative control. For 60S40C-CL and 60S36C4P-CL samples, the viability of cells after exposure to the material dissolution products were approximately 70% (Figure 8A). For 60S32C8P-CL and 60S28C12P-CL, viability was higher at approximately 87% (Figure 8B). Hence, all hybrids made in this work passed the ISO standard cytotoxicity test and did not induce cytotoxicity effect, so that they could potentially be used in biological applications. The increase in cell viability may be due to the slower dissolution rate and a buffering effect of phosphate dissolution. *In vivo* studies on 70S30C bioactive glass foam scaffolds needed preconditioning because the too fast dissolution of Ca ions produced a burst increase in pH (Midha et al., 2013). Here Ca^2+^ release from 60S40C-CL and 60S36C4P-CL is still compatible with cell viability, but the increase in P content is believed to have a beneficial effect in buffering the pH.

Cell attachment was examined by fluorescence confocal microscopy, following immunohistochemistry staining. In Figures 8C–F, green indicates tubulin, red is F-actin and blue shows the position of the nucleus. Figures 8C–F show that all Ca-containing hybrids supported good cell attachment and spreading, showing potential to be used as scaffolding materials in bone tissue engineering applications. Expression of tubulin on 60S40C-CL appeared more prominent than on other hybrids: this can be an indication of better cell migration, spreading and potentially differentiation (Schlunck et al., 2008; Ganguly et al., 2012). In addition, cells attached on 60S40C-CL and 60S36C4P-CL demonstrated better spreading than 60S32C8P-CL and 60S28C12P-CL. A number of material surface properties can affect cell attachment, including wettability, stiffness, surface structure etc. Different results were observed when Ca-free 100S-CL reference hybrid was tested for MC3T3 cell attachment (Supplementary Figure S3). Although cells appear viable on 100S-CL, the lack of adhesion spreading suggests MC3T3 cells would likely experience inferior proliferation and subsequent osteogenic differentiation on Ca-free samples, which could be due to the surface chemistry, or the lower stiffness of 100S-CL hybrid due to the lack of Ca content in the sample. These findings suggest that the addition of calcium imparts novel mechanical and osteogenic properties to SiO_2_/PTHF/PCL-diCOOH hybrid system.

## Conclusion

Calcium and phosphate were successfully incorporated into covalently-bonded silica-based hybrids containing PTHF and PCL as organic components (i.e., SiO_2_/PTHF/PCL-diCOOH) by using CME and TEP as the calcium and phosphate precursors respectively. Those additions made the inorganic part analogous to a bioactive glass containing 60 mol% SiO_2_, which imparted calcium ion release and HCA formation in SBF. The presence of calcium and phosphates improved preosteoblast cell attachment compared to calcium and phosphate-free hybrids, and resulted in a more congruent degradation of the inorganic and organic co-networks in TRIS buffer. All hybrids passed the ISO standard cell cytotoxicity test and supported good MC3T3-E1 (preosteoblast) cell attachment, even though cells cultured on P-free hybrids (i.e., 60S40C-CL) demonstrated better spreading and potentially differentiation than when phosphate was also added. The presence of calcium was beneficial to the mechanical properties, increasing the strength of the original SiO_2_/PTHF/PCL-diCOOH hybrid by one order of magnitude, maintaining a viscoelastic and tough behaviour with ability to resist cyclic loading, which is promising for application in bone; whereas, the addition of P reduced the stress at failure, which still remained five times higher than the stress at failure of the original SiO_2_/PTHF/PCL-diCOOH hybrid even at the highest investigated percentage of phosphates (i.e., 60S28C12P-CL). Therefore, it can be concluded that 60S40C-CL (i.e., SiO_2_-CaO/PTHF/PCL-diCOOH) is the most promising degradable, tough and bioactive hybrid composition for bone regeneration among the tested hybrids. This represents the first sol-gel inorganic/organic hybrid material that shows both outstanding mechanical properties and bone bioactivity, achieving the goal of increasing the toughness of bioactive glass maintaining its bone regeneration potential.

## Supplementary Material

Supplementary information

## Figures and Tables

**Table 1:** Nominal compositions of the inorganic component SiO_2_-CaO-P_2_O_5_/PTHF/PCL-diCOOH, produced by the substitution of SiO_2_-CaO-P_2_O_5_ for SiO_2_ in the SiO_2_/PTHF/PCL-diCOOH hybrid system developed by Tallia *et al*. (Tallia et al., 2018), and inorganic/organic ratio determined by DSC/TGA. All hybrid compositions were made starting from TEOS/PCL-diCOOH of 80/20 wt%, to which CME and TEP were added in order to obtain the corresponding SiO_2_/CaO/P_2_O_5_ molar composition in the inorganic component. The reference composition 100S-CL (*) is the Ca-free and P-free hybrid composition.

Sample ID	SiO_2_ (mol%)	CaO (mol%)	P_2_O_5_ (mol%)	I/O ratio (wt% from DSC/TGA)
100S-CL (reference*)	100	0	0	24.7/75.3
60S40C-CL	60	40	0	32.9/67.1
60S36C4P-CL	60	36	4	33.6/66.4
60S32C8P-CL	60	32	8	30.6/69.4
60S28C12P-CL	60	28	12	30.0/70.0

**Table 2:** Chemical shifts (obtained from ^29^Si CPMAS NMR) and percentage abundance (quantified from ^29^Si single-pulse MAS NMR) of silicon T and Q species and the corresponding D_c_ for the SiO_2_-CaO-P_2_O_5_/PTHF/PCL-diCOOH hybrids.

Sample ID	T^1^		T^2^		T^3^		Q^1^		Q^2^		Q^3^		Q^4^		D_c_ [%]
	*δ* [ppm]	*I* [%]	*δ* [ppm]	*I* [%]	*δ* [ppm]	*I* [%]	*δ* [ppm]	*I* [%]	*δ* [ppm]	*I* [%]	*δ* [ppm]	*I* [%]	*δ* [ppm]	*I* [%]	
Error	*(*± *0.2)*	*(*± *0.5)*	*(*± *0.2)*	*(*± *0.5)*	*(*± *0.2)*	*(*± *0.5)*	*(*± *0.2)*	*(*± *0.5)*	*(*± *0.2)*	*(*± *0.5)*	*(*± *0.2)*	*(*± *0.5)*	*(*± *0.2)*	*(*± *0.5)*	(± *1.0*)
**100S-CL**	-	-	-58.8	3.8	-66.0	14.5	-	-	-	-	-102.5	27.9	-111.5	53.8	91.8
**60S40C-CL**	-55.0	1.7	-61.3	5.7	-68.7	8.3	-84.3	1.0	-97.0	12.0	-104.6	21.1	-113.2	50.1	84.8
**60S36C4P-CL**	-54.1	2.3	-59.6	3.9	-66.0	11.0	-80.0	2.6	-92.9	15.6	-101.3	22.3	-110.3	42.3	81.8
**60S32C8P-CL**	-52.1	1.3	-60.5	5.2	-69.0	12.6	-82.4	2.7	-95.0	38.8	-103.6	15.5	-112.7	23.8	72.0
**60S28C12P-CL**	-	-	-60.4	7.9	-68.8	11.2	-82.9	0.4	-95.0	27.8	-104.3	24.5	-113.4	28.1	76.9

**Table 3:** Mechanical properties of hybrid monoliths calculated from uni-axial compression test to failure. Mean values ± standard deviation (n ≥ 3).

Sample ID	Strain at failure, ε_c_ (%)	Stress at failure, σ_c_ (MPa)	Modulus of toughness, U_T_ (MPa)
100S-CL	27.9 ± 3.3	3.3 ± 0.9	0.35 ± 0.10
60S40C-CL	55.9 ± 5.1	63.7 ± 20.9	14.0 ± 5.2
60S36C4P-CL	50.4 ± 3.1	34.8 ± 4.4	6.7 ± 1.0
60S32C8P-CL	48.2 ± 0.3	22.9 ± 1.0	4.0 ± 0.2
60S28C12P-CL	48.5 ± 3.2	16.8 ± 2.8	2.7 ± 0.4

**Table 4:** Weight loss of hybrids after 1 week of TRIS buffer dissolution and the variation in inorganic wt% in the hybrids before and after 1 week TRIS dissolution measured from TGA.

Sample ID	Weight loss (wt%)	Original inorganic content from TGA (wt%)	Inorganic content after 1 week in TRIS from TGA (wt%)
**60S40C-CL**	14.2	32.9	34.0
**60S36C4P-CL**	15.3	33.6	35.0
**60S32C8P-CL**	8.06	30.6	31.2
**60S28C12P-CL**	10.8	30.0	34.0

## Data Availability

The data that support the findings of this study are available from the corresponding author upon reasonable request from rdm-enquiries@imperial.ac.uk.
