# Supplementary material for: Bioactive, Degradable and Tough Hybrids Through Calcium and Phosphate Incorporation
Source: Front Mater. Author manuscript; Available in PMC 2024 Dec 8. (PMC7616990; doi:10.3389/fmats.2022.901196)
Supplement: Supplementary information [file EMS193201-supplement-Supplementary_information.PDF]

Supplementary Material

Supplementary Figures

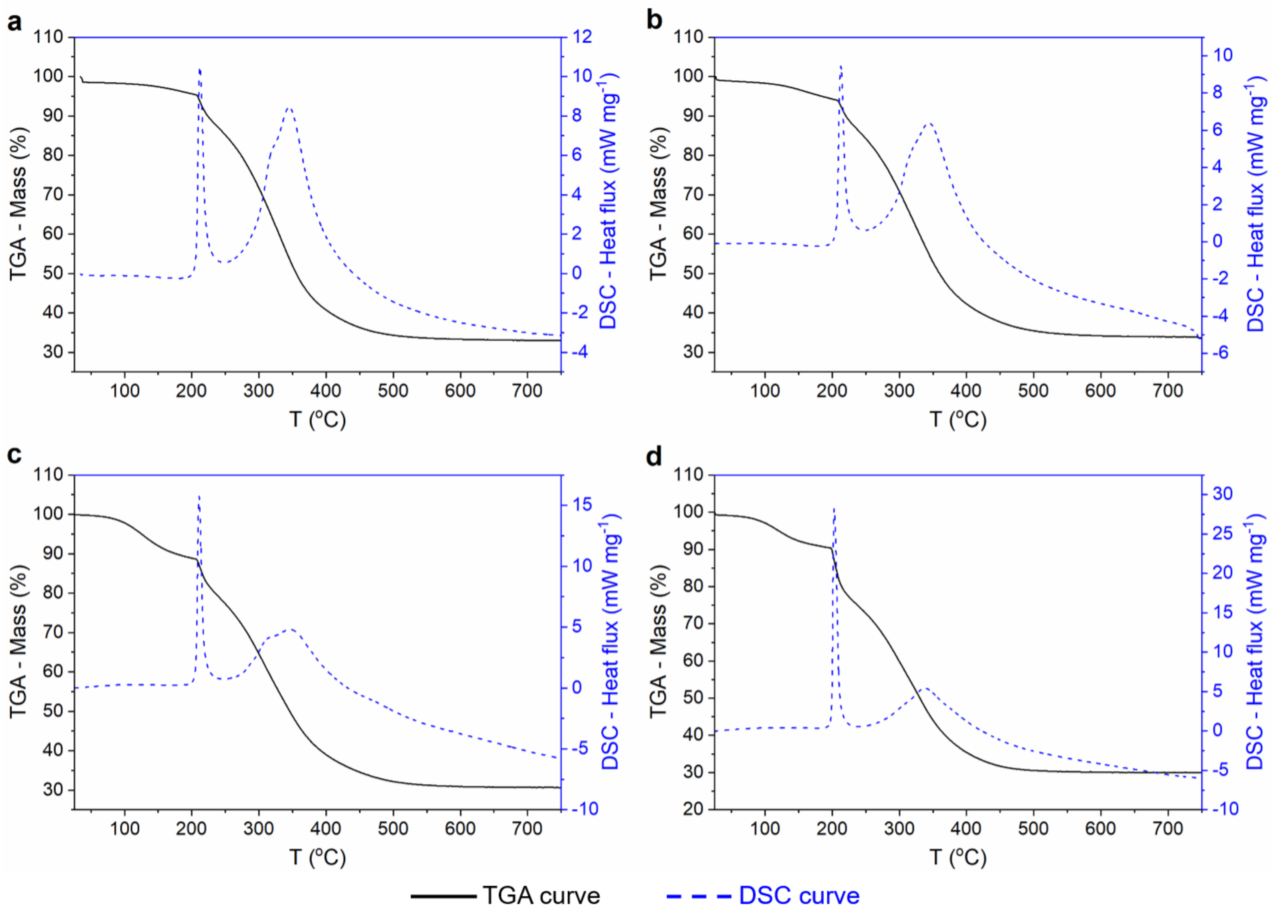

Supplementary Figure 1. TGA and DSC curves of (a) 60S40C-CL, (b) 60S36C4P-CL, (c) 60S32C8P-CL, (d) 60S28C12P-CL.

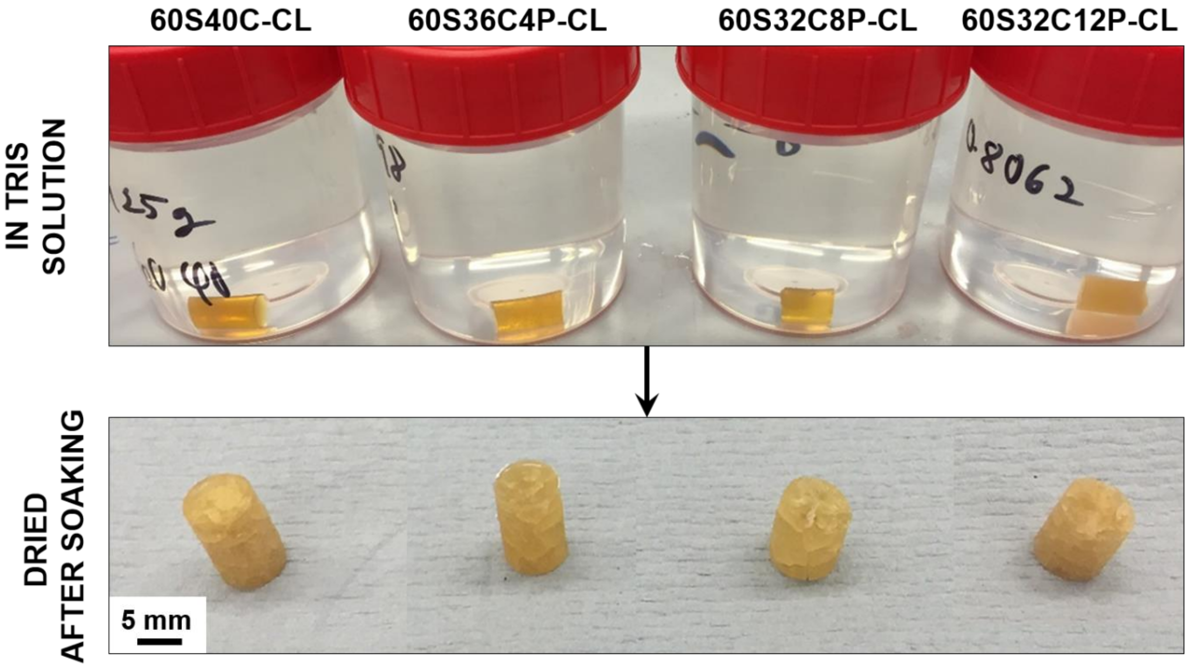

Supplementary Figure 2. Observations of behaviour of the four Ca-containing hybrids during and after immersion in TRIS solution.

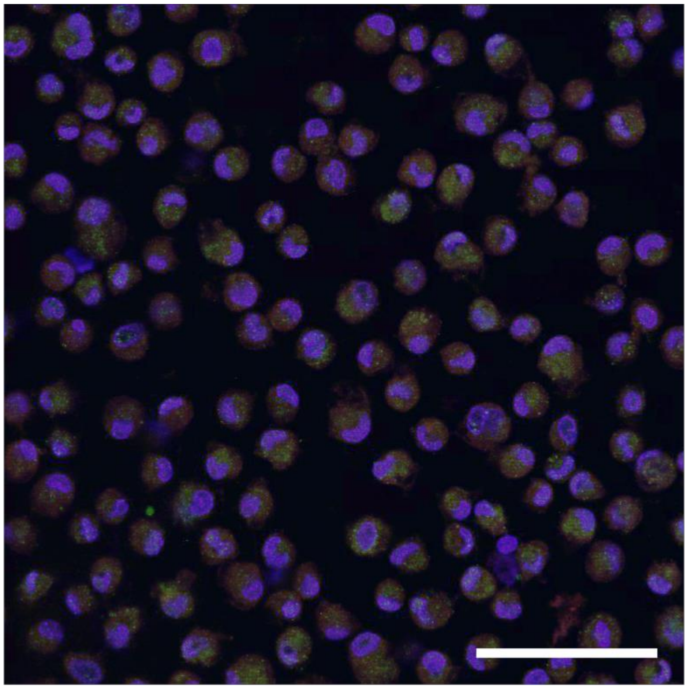

Supplementary Figure 3. Confocal microscopy images of the preosteoblast cells cultured on the surface of a Ca-free 100S-CL hybrid sample. Green indicates tubulin, red is F-actin and blue shows the position of the nucleus. Scale bar = 100 μm.
